# Review of "*The Globalisation Of Addiction: A Study In Poverty Of The Spirit*" by Bruce K. Alexander

**DOI:** 10.1186/1477-7517-6-12

**Published:** 2009-06-23

**Authors:** Harry G Levine

**Affiliations:** 1 Sociology Department, Queens College, City University of New York, USA

## Abstract

Book review of "*The Globalisation Of Addiction: A Study In Poverty Of The Spirit*" by Bruce K. Alexander

In a 1970s *New Yorker *cartoon, a writer at a small desk pecks away on a typewriter. In his thought cloud behind him, fifteen people cheer enthusiastically and say: *"A brilliant achievement ... Unflinching ... Writing at its most illuminating ... Explosive ... Long overdue ... True vision ... Plain speech ... Proclaims the failure of our civilization as a whole."*

*The Globalisation of Addiction *is that kind of book – and I mean that in the best and most ambitious sense. It earns each of those descriptions from "brilliant" to "proclaims the failure of our civilization." Among the book's major inspirations are works of Erik Erikson, Karl Polanyi, Eric Fromm, Emile Durkheim, Phillip Slater, and certainly Marx and Freud. There is something almost traditionally European about its combination of erudition, ambition, seriousness, and enormous scope. It is the result of a life's work reading, researching and thinking: 470 well-written pages with over a thousand end notes. Like those classic thinkers, Bruce Alexander has focused his attention on a central problem in the modern world, sought to describe it, contextualize it in large social, economic and historical terms, and contribute to seeing the way out.

*The Globalisation of Addiction*'s argument often makes surprising turns and explorations – each worth following for its information about the history of Western culture and about the contemporary world. Part of what is disorienting is the book's unusual combination of conventional, North American understandings – including its use of the word "addiction" – and its utterly unconventional and thoroughgoing expansion of such understandings across categories, cultures, and historical epochs. The book's major and secondary arguments are often simultaneously quite familiar and remarkably strange.

Alexander, a distinguished professor of psychology in Vancouver, Canada, differentiates four main types of "addiction." But he focuses on one particular meaning of addiction which he defines and restates throughout the work. This meaning incorporates (but is not limited to) what most people have in mind when they think of someone seriously addicted to alcohol, heroin, or cocaine. It includes what members of Alcoholics Anonymous mean as well as what most physicians, psychologists, and drug treatment professionals mean – most simply an "overwhelming involvement" with drugs that harms the person or others. Like most people, Alexander regards severe addiction as painful, destructive, and tragic. Unlike many health professionals in North America, however, Alexander does not regard addiction as primarily a medical condition and certainly not a disease, and he argues that modern, scientific medicine (despite its many successes with other chronic conditions) has been spectacularly unsuccessful at curing or preventing drug addiction.

But Alexander does not stop there, or more precisely he just begins there. The meaning or type of addiction this book focuses on is not primarily addiction to drugs. It also refers to "overwhelming" and harmful involvements "with any pursuit whatsoever." And this means, he says, addiction to: "Gambling, love, power-seeking, religious or political zeal, work, food, video game playing, internet surfing, pornography seeking," shopping, and much more. These addictions can take up every aspect of a person's life: "conscious, unconscious, intellectual, emotional, behavioural, social and spiritual – just as severe drug and alcohol addiction can." Sometimes Alexander accepts the vocabulary of conventional drug treatment and recovery programs. " Such overwhelming involvements," he says, "often entail a startling blindness to the harm that the addiction is doing, which is aptly called 'denial'." But Alexander expands the phenomena covered by this and suggests that "many instances of addiction do not involve a single habit, but rather an 'addictive complex' of several habits that constitute a single addictive lifestyle."

Alexander says that all such addictions exist on a continuum of severity, from a mild problem that "only occasionally overwhelms a person's life" and may be short-lived, to the middle of the continuum where addicted people "strive to maintain a double life" and the appearance of normality, to the severe end when the addiction cannot be concealed, destroys the person's conventional lifestyle, causes great harm to others, and "can reach an unrelenting, hellish intensity and may have fatal consequences."

*The Globalisation of Addiction *is about all forms of harmful addictions – the relatively small number centered on drugs and the great many more addictions that have nothing to do with drugs. As the title suggests, Alexander sees these addictions increasing dangerously throughout the world. But he also sees them as a recurring feature of Western civilization and to some extent of all large civilizations.

Among the book's more surprising and intriguing turns is its examination of addiction (in this broad sense) in writings from other times and cultures, including from ancient civilizations. For example, Alexander focuses on writings of Augustine of Hippo, the great 4th century theoretician of early Christianity, and of Plato, the great philosopher of ancient Greece. By examining both Augustine and Plato, Alexander in effect raises the enduring problem of the disruptive power of human desires and appetites, for individuals and for the people around them. The Greeks and early Christians both thought much about this and discussed the anti-social potential of human appetites. By quoting and explicating their words, Alexander drops us into utterly foreign cultures where people are talking in surprisingly familiar ways about "addiction" – though of course without using that word. And he does the same with the life of James M. Barrie, the talented and extremely odd author of *Peter Pan*. In all these cases and others, including China and his own city of Vancouver, Alexander moves easily among very different cases of what might otherwise be called "obsessions" or "compulsions" (However, Alexander does not use either word much.) For Alexander, and for the reader who follows along, these are all addictions.

Viewing ancient and other discussions of great, persistent, obsessive desire as "addiction" is an unusual and radical idea. But as Alexander shows, it is surprisingly effective and useful for clearing intellectual clutter and seeing beyond conventional views.

First of all, this major expansion of what gets included as "addiction" totally undermines the claims of chemical or pharmacological determinism – the popular idea that certain substances like alcohol or heroin possess unique addictive or "enslaving" powers. For many years, gambling addiction has served that debunking function because gambling addicts and members of Gamblers Anonymous reported the same kind of cravings, binges, loss-of-control, ups, downs, and even withdrawals as alcohol and other drug addicts, *without a drug. *Food and sex and other twelve step addiction programs eventually did so as well.

But once one introduces Augustine talking about his enslavement to lust and women, or Socrates talking about a man whose "best elements" are "enslaved and completely controlled by a minority of the lowest and most lunatic impulses" – once they are introduced as discussing real-life, genuine, familiar, present-day style addiction – then claims about the supposed, unique, addictive powers of a few substances seem rather silly and beside the point. And in one chapter, Alexander provides a masterful debunking of "The Myth of Demon Drugs." This expansion of addiction far beyond drugs also takes the ground out from under arguments about why some addictions should be prohibited and criminalized, while other more common and equally pernicious ones are not.

Second, this radical expansion of addiction irrevocably moves addiction away from scientific medicine and treatment and puts it instead in the tradition of discussions of the great dilemmas of life and civilization. In important ways, with addiction as the focus, Alexander is taking on at least part of Freud's questions in *Civilization and Its Discontents*, Fromm's in *Escape From Freedom*, and similar civilization-wide explorations of the paradoxes of the human condition. In effect Alexander argues that properly understood as an enduring human problem, addiction opens a new window on the human condition – in our globalizing, consumerist, nuclear-bomb loaded, environmentally-disintegrating 21st century.

Third, this radical expansion allows Alexander to develop his central thesis about what addiction is and what increases its likelihood. And, alternatively, what addiction is not and what decreases addiction's likelihood. He argues that addiction is an individual and social response to "dislocation" – especially severe social, economic, and cultural dislocation. The alternative or opposite of dislocation is "psychosocial integration," a conception he builds upon from Erik Erikson and others. As Alexander explains:

"Psychosocial integration is a profound interdependence between individual and society that normally grows and develops throughout each person's lifespan... Psychosocial integration is experienced as a sense of identity because stable social relationships provide people with a set of duties and privileges that define who they are in their own minds.... Psychosocial integration makes human life bearable and even joyful at its peaks. Moreover it is a key to the success of the human species, which flourished by simultaneously evolving close cooperation and individual creativity."

"Lack or loss of psychosocial integration was called 'dislocation' by Karl Polanyi. Dislocation ... denotes psychological and social separation from one's society, which can befall people who never leave home, as well as those who have been geographically displaced. Like psychosocial integration, dislocation has been given many names, perhaps the most familiar being 'alienation' or 'disconnection' ...."

It is this understanding of the powers of dislocation that is captured in the subtitle of this book: "A Study In Poverty Of The Spirit."

"People can endure dislocation for a time. However, severe, prolonged dislocation eventually leads to unbearable despair, shame, emotional anguish, boredom and bewilderment. It regularly precipitates suicide and less direct forms of self-destruction. This is why forced dislocation, in the form of ostracism, excommunication, exile, and solitary confinement, has been a dreaded punishment from ancient times until the present...."

"Material poverty frequently accompanies dislocation, but they are definitely not the same thing. Although material poverty can crush the spirit of isolated individuals and families, it can be borne with dignity by people who face it together as an integrated society. On the other hand, people who have lost their psychosocial integration are demoralized and degraded even if they are not materially poor. Neither food, nor shelter, nor the attainment of wealth can restore them to well-being. Only psychosocial integration itself can do that. In contrast to material poverty, dislocation could be called 'poverty of the spirit'."

Dislocation can have many causes and has been more severe at different times and circumstances. Alexander skillfully takes the reader through numerous cases of this broadly conceived idea of addiction, locating each in specific, well-explained situations of dislocation – from Augustine's *Confessions *to transcripts with junkies he has interviewed in Vancouver. For Alexander, addiction is always best understood as a response to dislocation. Indeed, the book could have been titled "Addiction and Dislocation."

But Alexander has more to say: he argues that addiction is an *adaptation *to dislocation. It is a *functional *way of responding to and dealing with dislocation. It is even a creative response that, for a while, can reduce the pain of dislocation. Whether with drugs or not, it is a kind self medication. However, for addicted individuals and for people around them, it is an adaptation that does not work well over time, and is often very harmful causing much suffering. For Alexander, addicts are people struggling to adapt to and deal with difficult psychological and social circumstances. Viewing addictions (again, of all kinds) as adaptive responses to dislocation seems odd at first – because we have been taught to view addiction in the narrow, conventional sense of magical, evil drug molecules taking over the brain. But viewing addictions as adaptations makes addiction both more comprehensible and more familiar, and is ultimately a deeply sympathetic and humane perspective. And it is a hopeful one, offering a variety of options that can help addicted individuals find social integration and therefore happier lives.

Finally, Alexander insists that the rapidly-expanding, modern, free-market, global capitalist system is a kind of super hothouse for the creation of every sort of dislocation, and therefore inevitably of all kinds of addiction. He stresses the disruptive, dislocating and even disintegrating powers of capitalist development. Like every other serious scholar of capitalism, Alexander learns from Marx – especially the famous passage where Marx poetically captures the revolutionary changes capitalism brings:

"Constant revolutionizing of production, uninterrupted disturbance of all social conditions, everlasting uncertainty and agitation distinguish the bourgeois epoch from all earlier ones. All fixed, fast frozen relations, with their train of ancient and venerable prejudices and opinions, are swept away, all new-formed ones become antiquated before they can ossify. All that is solid melts into air, all that is holy is profaned "

It seems to me that much of Alexander's argument about the dislocating effects of free market capitalism is a thoughtful extension to the present of this understanding. And in 2009, deep into the biggest world-wide economic catastrophe since the Great Depression of the 1930s – with banks, businesses, jobs and savings being swept away – I think this is not a hard point to understand. In this regard, the book has great timing.

Although his scope is global and spans several millennia, Alexander begins and ends his book with the street junkies and addicts of Vancouver. For decades, Alexander has worked hard for drug policy reform and harm reduction, and his heart is with the lowliest junkies and addicts. He understands they need a range of services – housing, employment, medical services, counseling – to help with their myriad economic and social problems. But he insists they absolutely need community, belonging, usefulness and positive group identities – they need social integration. And his concluding chapter offers ways of thinking about policies that help provide integration – to reduce the likelihood that people will seek refuge in addiction, and to increase the chances they can turn away from it.

Since the 1970s, a number of historical researchers have concluded that the present-day understanding of drug addiction – as overwhelming desire for and uncontrollable use of psychoactive drugs – is a modern idea, first emerging in popular thought in the early 19th century in North America. Alexander has put forth the bold, challenging proposition that this has actually been a renaming of a much older and larger human problem, one that is now increasing throughout the world. He suggests that when understood that way, what we call addiction can be viewed far more sympathetically and effectively. In years to come, *The Globalisation of Addiction *will likely provide a starting place for much fruitful research and theorizing. Hopefully it will also inspire more humane politics and policies. It is, indeed, a brilliant achievement.

A 2001 paper by Professor Alexander [1] that discusses some of the circumstances and history of the Four Pillars approach in Vancouver, providing the basis of the book, may be of interest to readers.
